# Enhanced Formation of Dicarboxylic Acids in the Catalytic Oxidation of Polyethylene With O_2_ and NO_x_

**DOI:** 10.1002/anie.6681773

**Published:** 2026-06-26

**Authors:** Tom J. Smak, Rinke Altink, Ina Vollmer, Bert M. Weckhuysen

**Affiliations:** ^1^ Inorganic Chemistry and Catalysis group Institute for Sustainable and Circular Chemistry Department of Chemistry Faculty of Science Utrecht University Utrecht the Netherlands; ^2^ TNO Brightsite Geleen the Netherlands

**Keywords:** catalysis, diacid, oxidation, polyethylene, recycling, transition metal ion

## Abstract

A promising strategy to valorize polyethylene is its oxidation to dicarboxylic acids with O_2_ and NO_x_. However, it remains unclear whether and how the formation of dicarboxylic acids from polyethylene can be increased through catalyst design and reaction parameters adjustment. In this work, it was found that through proper reaction condition selection and in the absence of a catalyst the yield toward dicarboxylic acids could reach ∼29 mol% with only little overoxidation to undesired CO and CO_2_. At high NO partial pressure, the formation of gaseous products is suppressed, yielding an excellent carbon recovery for a variety of polyethylene types. The yield toward dicarboxylic acids can be further enhanced through the addition of a Cu^2+^/V^5+^ catalyst up to ∼51 mol% (>90% wt.% diacid), while the excellent carbon recovery can be maintained. It was found that the catalyst system was both robust and tolerant as the use of post‐consumer plastic waste, a mixture of polyethylene and polypropylene, yielded a similar product slate.

## Graphical abstract

The oxidation of polyethylene in the presence of a mixture of O_2_ and NO_x_ and with Cu^2^^+^/V^5^^+^ as catalyst yields ∼51 mol% diacids, while retaining ∼80 mol% of the carbon in the oil fraction. The developed method is robust and tolerates several polymer types as well as impurities.

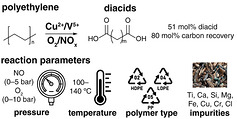

## 1 Introduction

With only 10% of all plastic waste ever produced being recycled, leading to environmental problems, including the formation of micro‐ and nanoplastics, the recycling rates should be improved [1]. In addition, we want to gradually move away from the current fossil based economy and move toward a more circular economy and keep the carbon in the loop, as recently outlined for designing the so‐called refinery of the future [2]. In recent years, the incentive for introducing chemical and physical recycling routes has grown and hence recycling rates have gone up, but the available technologies are still far from optimal [3, 4, 5]. A promising method to valorize polyethylene (PE) waste is selective oxidation. These methods involve either post‐modification of the PE backbone [6, 7] or the conversion toward high‐value dicarboxylic acids, such as succinic and adipic acid [8, 9]. These dicarboxylic acid monomers are used for the production of polymers, such as nylon‐6,6 [10] and polybutylene succinate (PBS) [11]. In addition, recent publications have shown that mixed dicarboxylic acids show potential to be used in polymer applications directly [12, 13].

**FIGURE 1:**
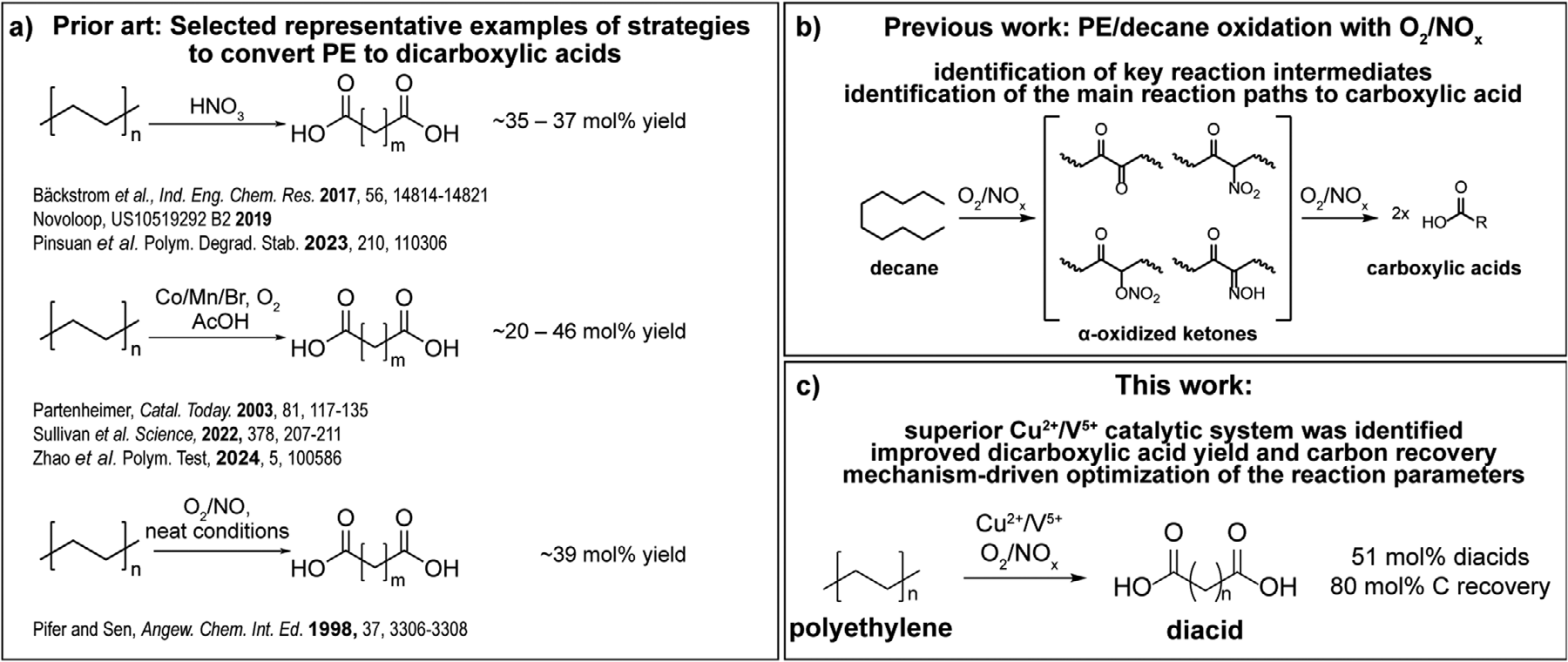
(a) In the literature several strategies have been reported to convert polyethylene (PE) into dicarboxylic acids. The higher yielding methods involve conditions using HNO_3_, Co/Mn/Br or O_2_ and NO_x_. (b) In previous work on the oxidation of PE and decane, key reaction intermediates were identified and main reaction paths were unraveled [20]. (c) In this work, the dicarboxylic acid yield and carbon recovery are increased by the addition of a Cu^2+^/V^5+^ catalyst and by selecting proper reaction parameters.

Over the past few years, the number of reported methods for converting PE into dicarboxylic acids has increased rapidly. An overview of the most relevant and noticeable strategies is provided in Figure 1a. As a detailed discussion of all examples is beyond the scope of this introduction, an expanded overview with additional examples and details is included in Section S1. A direct comparison, between all the reported methods is challenging due to the variation of the methods and how the product yield is reported. Often product yields are reported based on wt.% instead of mol%. In addition, other factors, such as normalization protocols, make a direct comparison difficult. In order to make a more fair comparison, the product yields have been converted from wt.% to mol%‐based yields for some examples. The mol%‐based yield is defined as the percentage of carbon from the original PE material that is retained in the dicarboxylic acid products.

Noticeable examples to produce dicarboxylic acids from PE include methods under conditions comparable to the Amoco Mid‐Century process (Co/Mn/Zr/Br in acetic acid, 180°C, and 70 bar air) [9, 14, 15], as well as methods involving stoichiometric amounts of HNO_3_ (Figure 1a, detailed description of all examples in Section S1) [16, 17, 18]. Both methods yield ∼40 mol% dicarboxylic acids [9, 14, 16, 17]. A promising alternative that combines advantages of both the Amoco Mid‐Century and HNO_3_‐based methods was reported by Pifer and Sen [8, 19]. They demonstrated PE conversion with a mixture of O_2_ and NO, yielding succinic, glutaric, adipic, and pimelic acids in a combined dicarboxylic acids yield of 39 mol%. The use of O_2_ and NO is particularly attractive because the reaction proceeds under neat conditions, employs O_2_ as the terminal oxidant, and avoids the need for expensive metal catalysts. All the above‐mentioned examples report dicarboxylic acid yield with low quantities of side products, while partially converted polymers and the gaseous products are not studied.

The initial communication by Pifer and Sen only provided one set of reaction conditions, and the underlying reaction mechanism remained unclear. In our previous work, the reaction pathways from alkanes to carboxylic acids using O_2_/NO were elucidated (Figure 1b). Using decane as a model compound for PE, we observed that carboxylic acids are formed through multiple steps involving a wide range of (nitrogenated) reaction intermediates [20]. In the C─C bond cleavage step, α‐derivatized ketones bearing an additional ketone, nitro, nitrate, and oxime group were experimentally detected. The identification of these intermediates is interesting, because they serve as a target for the development of a catalytic system. Literature suggests that the carboxylic acid yields in the C‐C cleavage step can be enhanced by the addition of a catalyst [21]. For the oxidation with O_2_/NO_x_, it remains unclear whether and how product formation can be increased through the proper selection of the reaction parameters.

In this work, we demonstrate that the yield toward dicarboxylic acids can be increased from ∼29 to ∼51 mol% (>90% wt.%) by the addition of a Cu^2+^/V^5+^ catalyst (Figure 1c). The effects of catalyst addition and proper reaction parameters selection on both dicarboxylic acids yield and carbon recovery were investigated using a combination of gas chromatography‐mass spectrometry (GC‐MS), gas chromatography‐flame ionization detection (GC‐FID), CHN analysis, and infrared (IR) spectroscopy. The GC‐MS/GC‐FID analysis allowed qualitative and quantitative analysis of the formation of dicarboxylic acids. Reaction intermediates present in partially oxidized PE were followed with IR spectroscopy, while CHN analysis yielded the total carbon recovery. In our previous mechanistic study on decane conversion, the same product analysis methods were used to study various time points in the reaction [20]. These results were compared to the results obtained from our current study on PE conversion, which allowed us to rationalize the observations made for PE. The parameters that were taken into account include the presence of Cu^2+^/V^5+^, temperature, NO pressure, O_2_ pressure, plastic type, as well as the presence of different relevant plastic waste impurities.

## 2 Results and Discussion

### 2.1 Effect of Reaction Parameters on the Oxidation of Polyethylene

**FIGURE 2:**
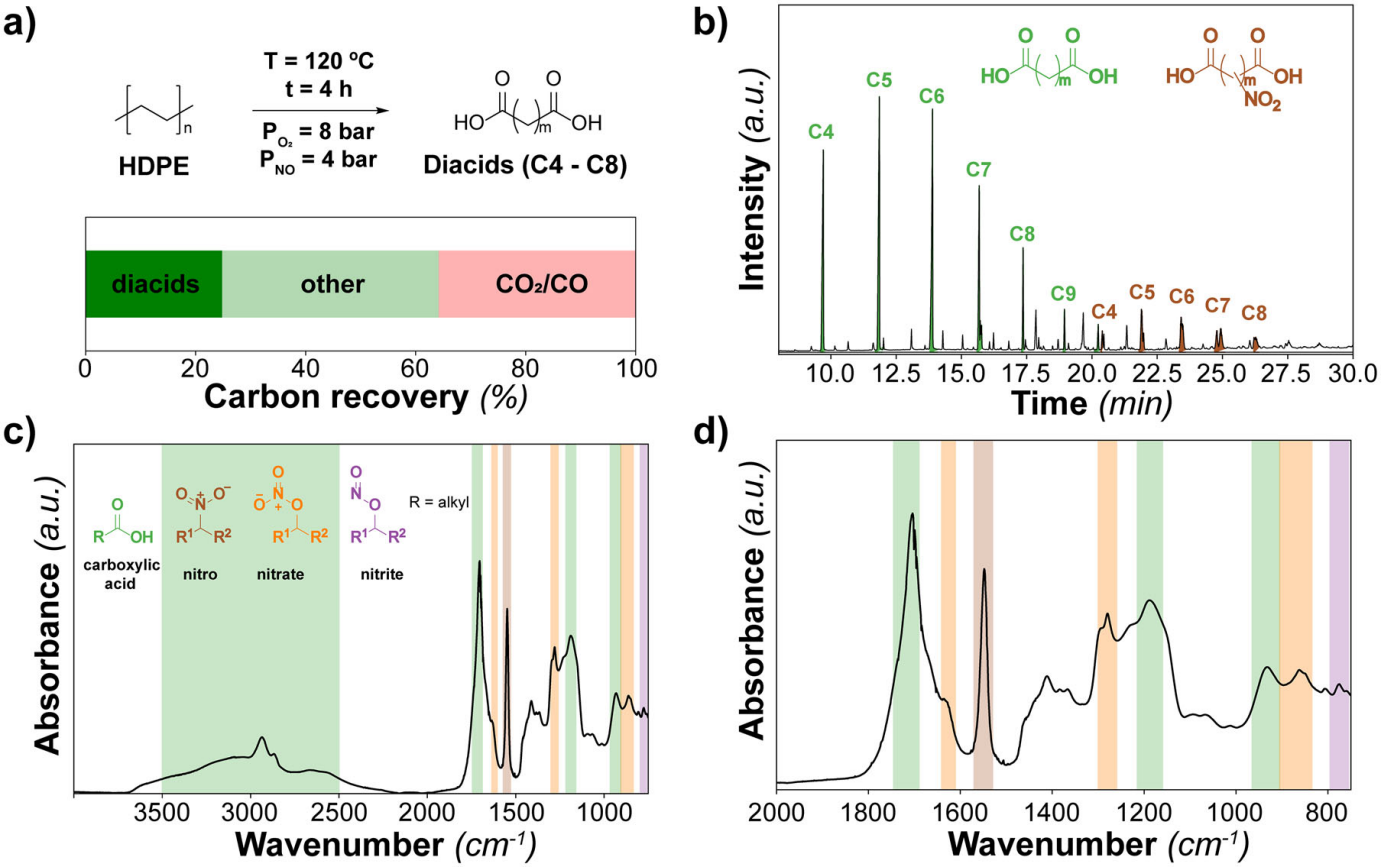
(a) High‐density polyethylene (HDPE) oxidation under standard conditions (i.e., T = 120°C, *t* = 4 h, P_O2_ = 8 bar, P_NO_ = 4 bar, balance N_2_, and P_total_ = 50 bar) produces a mixture of dicarboxylic acids, oligomers and CO_2_/CO. (b) Gas chromatography‐flame ionization detection (GC‐FID) chromatogram of HDPE oxidized under standard conditions. (c) Infrared (IR) spectrum of the nongaseous product mixture. (d) Zoom‐in of (c).

The effect of the reaction parameters on the reaction mechanism was studied to investigate whether the diacids yield and carbon recovery could be substantially increased. The PE oxidation experiments were performed batchwise in a 50 mL Parr autoclave. Most experiments were performed with 200 mg of high‐density polyethylene (HDPE) with a mass average molecular weight (M_w_) of 137 500 g/mol. When PE was subjected to an oxidative atmosphere of O_2_/NO_x_ under standard conditions in the absence of a catalyst (i.e., 120°C, 4 h, P_NO_ = 4 bar, and P_O2_ = 8 bar; Figure 2a), a transparent colorless oil was typically obtained, except at very low conversions (polymer‐like solids) and very harsh conditions (brown/black solids). An interesting property of the oil is that it has a low viscosity, allowing for other processing steps. A qualitative and quantitative analysis of the smaller cleavage products was performed using GC‐MS/GC‐FID [22, 23]. Prior to GC analysis the dicarboxylic acids were esterified to their methyl ester analogues. In a typical GC‐FID chromatogram a distribution of diacids ranging from C4 – C10, is obtained (Figure 2b). The yields are defined as the amount of carbon from PE that ends up in the product. An example calculation can be found in Section 4. Under standard conditions, dicarboxylic acids and nitro dicarboxylic acids account for ∼25 mol% and ∼5 mol%, respectively. The amount of carbon retained in the oil fraction was determined with elemental (CHN) analysis (example calculation in Section 4). Under standard conditions, the carbon recovery was determined to be ∼64 mol%, which is significantly more than what could be analyzed with GC. In most experiments, a relatively large fraction of partially oxidized polymeric or oligomeric and thus non‐volatile product remained.

The entire oil fraction including the nonvolatile products was characterized with IR spectroscopy (Figure 2c,d). Peaks corresponding to carboxylic acids were observed between 3500 and 2500 cm^−1^ (O‐H stretch), 1710 cm^−1^ (C═O stretch), 1240 cm^−1^ (C‐O stretch), and 940 cm^−1^ (O‐H bend) [24]. The carboxylic acid signals contribute most to the total intensity of the IR spectrum, which is in line with the observations made with GC. In addition, at 1550 cm^−1^ a peak assigned to the nitro group was observed (N═O asymmetric stretch) [24]. The strong absorbance of this peak, which is comparable to what has been observed in decane models [20], indicates that the non‐volatile product mainly bears nitro groups on the backbone. Furthermore, nitrate groups were observed with peaks at 1625 cm^−1^ (N═O asymmetric stretch), 1280 cm^−1^ (N═O symmetric stretch) and 870 cm^−1^ (N‐O stretch) [24]. At last, in some samples small quantities of the more reactive nitrite species were observed at 780 cm^−1^ (N‐O stretch) [24]. Other species that are expected to be present, such as alcohols and ketones, were not observed, presumably due to their low concentration. All the above mentioned assignments are in line with our previous study for the oxidation of decane [20].

The amounts of carbon not found in the solid/liquid residue are oxidized gaseous carbon products that were analyzed with IR spectroscopy (Section S2). Gas phase analysis showed the formation of CO_2_ and to a minor extent also the formation of CO. In addition, NO_2_ and N_2_O are present, while NO is not observed. This suggests that N_2_O is produced during the reaction. Additional analysis of the reaction gas before reaction only showed NO_2_ and N_2_O_4_, suggesting that NO is quickly oxidized to NO_2_ under reaction conditions.

**FIGURE 3:**
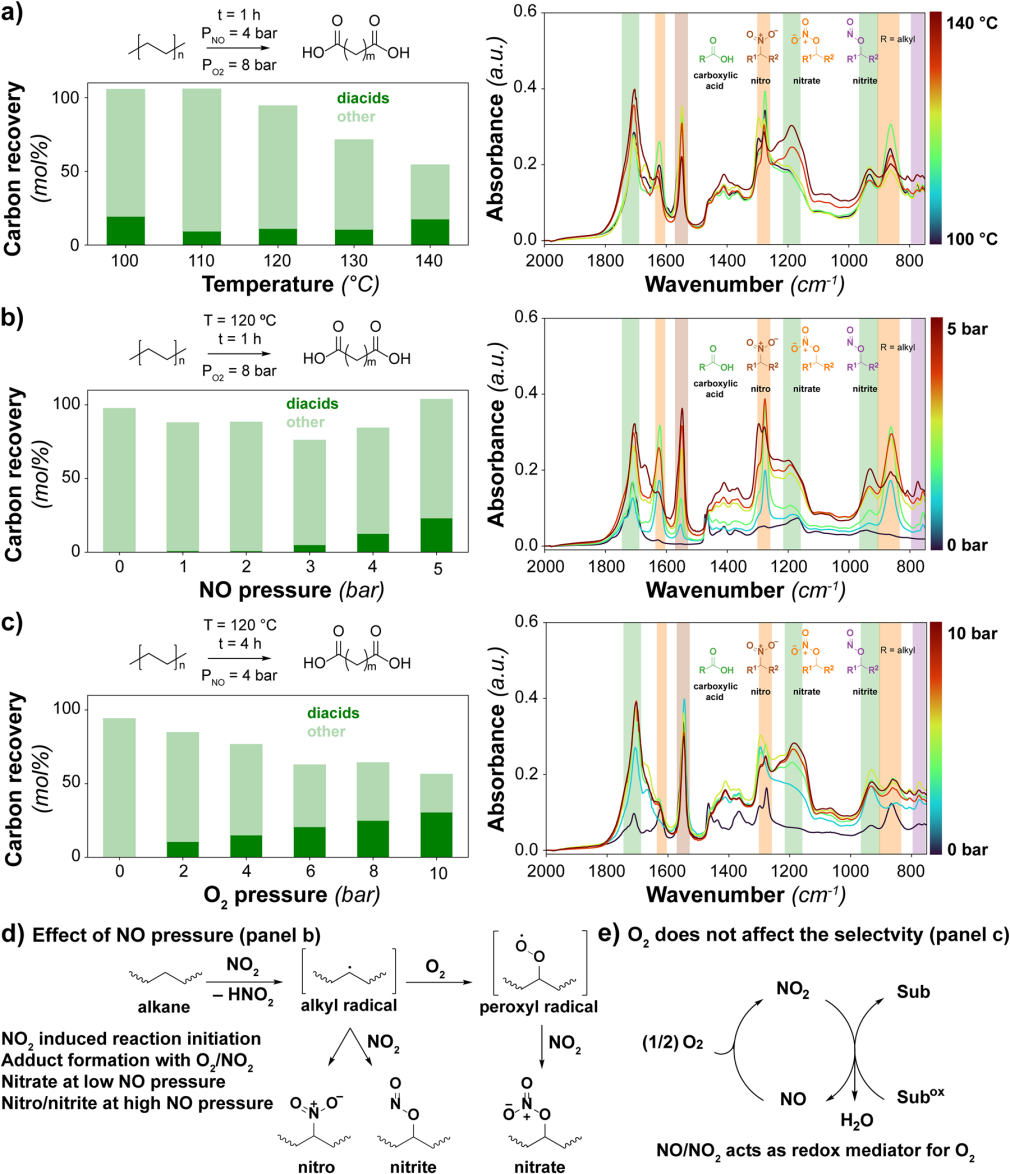
(a) Dicarboxylic acids yield, carbon recovery, and infrared (IR) spectra of oxidized high‐density polyethylene (HDPE) as function of temperature. HDPE was oxidized for 1 h at the following gas pressures, P_NO_ = 4 bar, P_O2_ = 8 bar, balance N_2_, and P_total_ = 50 bar. (b) Dicarboxylic acids yield, carbon recovery, and IR spectra of oxidized HDPE as function of NO pressure. HDPE was oxidized for 1 h at 120°C with the following gas pressures, P_O2_ = 8 bar, balance N_2_, and P_total_ = 50 bar. (c) Dicarboxylic acids yield, carbon recovery, and IR spectra of oxidized HDPE as function of O_2_ pressure. HDPE was oxidized for 4 h at 120°C with the following gas pressures, P_NO_ = 4 bar, balance N_2_, and P_total_ = 50 bar. (d) The effect of the NO pressure on reaction initiation and adduct formation. (e) With increasing O_2_ pressure, the reaction rate increases, but the selectivity remains similar. This means that O_2_ mainly regenerates reduced NO_x_ species.

In our previous work, we found that the dicarboxylic acids yield plateaus at ∼29 mol% with longer reaction time (120°C, >4 h) [20]. When the reaction is performed for longer than 4 h, the amount of dicarboxylic acids remains stable, but the carbon recovery continues to decrease. This suggests that there is a point where carboxylic acids are decomposed to CO_2_/CO at a similar rate as they are produced, but also independent other reaction pathways can play a role. Nevertheless, an optimum exists where a good amount of diacids yield is obtained without producing too much gaseous products. It has to be noted however, that although full carbon recovery is desired for circularity, the overall sustainability of the process might benefit from a higher overall selectivity to carboxylic acids even at the expense of forming CO_2_ and CO, as it would lower overall separation cost. This will have to be investigated in future work. While the focus of our previous study was the reaction time, in this section, we studied the effect of reaction temperature, NO pressure and O_2_ pressure (Figure 3).

With increased temperature at 1 h reaction time (Figure 3a), the dicarboxylic acids yield barely changes, going from 19 to 17 mol%. IR spectroscopy revealed only small differences in the residual oil composition with a slightly larger contribution of nitrate groups at lower temperatures. However, a more pronounced effect was observed regarding the overall carbon balance. Barely any gaseous products were formed at 100°C–110°C, while more than 46 mol% gas is formed at 140°C.

A large variation in diacids yield, carbon recovery, but also in physical appearance of the sample was observed when the NO pressure was varied from 0 to 5 bar (Figure 3b). In the absence of NO, the product remained largely PE‐like, showing only slight softening and yellowing, indicative of a slow reaction. GC analysis revealed only minor product formation, comparable to aerobic auto‐oxidation of PE [25]. At low NO pressures, the reaction proceeded faster than aerobic oxidation, but the dicarboxylic acids yield remained low and the carbon recovery slightly decreased. In addition, at low NO pressure nitrate groups dominate, while at 5 bar, nitro groups appeared alongside nitrates. This shift suggests that at low NO_x_ levels, alkyl radicals preferentially react with O_2_, while at higher NO_x_ levels radical adduct formation with NO_2_ becomes dominant (Figure 3d) [20]. At higher NO pressure both diacids yield and carbon recovery improved. At 5 bar NO, a yield of ∼23 mol% toward diacids was achieved with nearly complete carbon recovery, demonstrating the potential of the O_2_/NO_x_ route to deliver high yields. This performance could be further enhanced by integrating separation strategies to recycle partially oxidized PE [20]. Similar trends were observed at extended reaction times (Section S3).

Keeping the NO pressure at 4 bar, and increasing the O_2_ pressures, a higher yield toward dicarboxylic acids and a lower carbon recovery was obtained (Figure 3c). In addition, IR spectroscopy reveals an increase in the degree of oxidation, while evolution of the spectral shape is comparable to what has been observed with increasing the reaction time [20]. This suggests that in the presence of NO, the reaction simply proceeds faster at higher O_2_ pressures, while the selectivity remains unchanged. In our previous work [20], we observed that O_2_ can form radical adducts with alkyl radicals leading to the formation of peroxyl radicals, that react further to nitrates in the presence of NO_2_. IR spectroscopy shows a comparable amount of nitrate groups at every O_2_ pressure, suggesting that the influence of O_2_ pressure on this species is small. This means that the reaction selectivity is determined by the more reactive NO_x_ derivatives (most probably NO_2_) that can serve as the oxidant [26]. Therefore, we conclude that the main role of O_2_ is to regenerate the reduced NO_x_ derivatives (Figure 3e) [27].

Out of all reaction parameters studied the NO pressure is the most important one, that is, both dicarboxylic acids yield and carbon recovery are higher at higher NO pressure. In addition, a higher carbon recovery can be obtained by lowering the reaction temperature and rate can be enhanced at higher O_2_ pressures.

### 2.2 Effect of Polymer Type and Impurities

**FIGURE 4:**
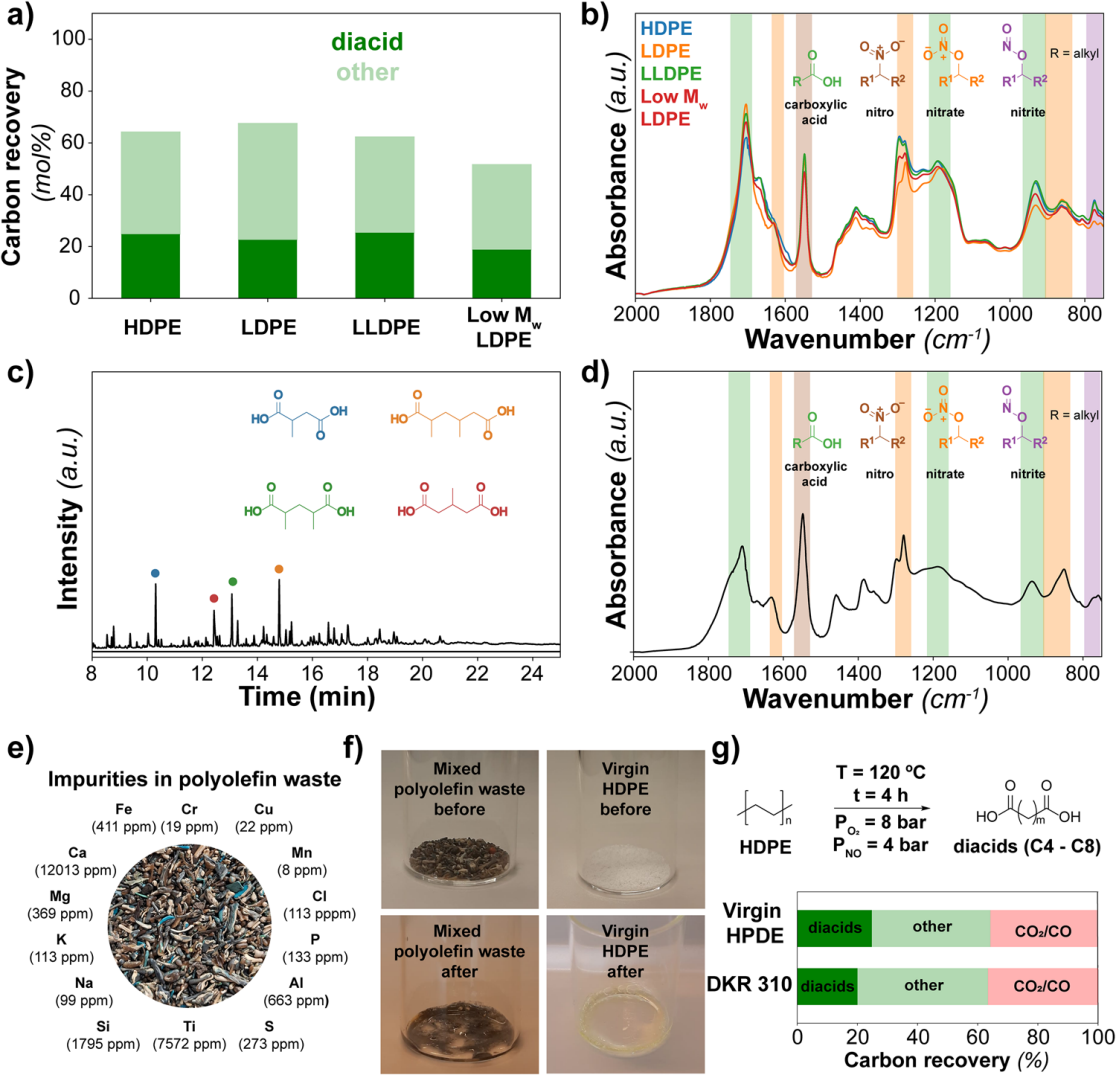
(a) Carbon recovery and dicarboxylic acids yield for the conversion of different polymer types, namely, high‐density polyethylene (HDPE), low‐density polyethylene (LDPE), linear low‐density polyethylene (LLDPE), and low molecular weight (M_w_) LDPE. The polymers were oxidized at 120°C for 4 h with P_NO_ = 4 bar and P_O2_ = 8 bar. (b) Infrared (IR) spectra of the oil fractions obtained under the same conditions as results presented in panel (a). (c). Gas chromatography‐flame ionization detection (GC‐FID) chromatogram of the products obtained from polypropylene (PP) subjected to oxidative conditions (i.e., 120°C, 2 h, P_NO_ = 4 bar, and P_O2_ = 8 bar). (d) IR spectrum of the oil fraction belonging to panel (c). (e) Impurities present in the polyolefin waste sample (DKR 310). (f) Pictures of the contaminated post‐consumer waste sample and virgin HDPE before and after the reaction. (g) The product distributions of both samples after the reaction performed at standard conditions (i.e., 120°C, 4 h, P_NO_ = 4 bar, and P_O2_ = 8 bar).

PE with varying degree of branching and M_w_ were used to study how the method would perform for different waste streams. The following PE types were selected: HDPE (M_w_ = 137500 g/mol), low‐density polyethylene (LDPE, M_w_ = 80980 g/mol), linear low‐density polyethylene (LLDPE, M_w_ = 52021 g/mol), and a low M_w_ LDPE (M_w_ = 4000 g/mol) that is frequently used in academic studies. The PE materials were oxidized under the standard conditions presented in Figure 2 (i.e., T = 120°C, *t* = 4 h, P_O2_ = 8 bar, P_NO_ = 4 bar, balance N_2_, and P_total_ = 50 bar), and for all PE types a comparable dicarboxylic acids yield (∼25 mol%) and carbon recovery (∼60 mol%) was obtained (Figure 4a). This means that more branching (∼2%) and lower M_w_ do not affect the outcome compared to HDPE. These observations were further corroborated with IR spectroscopy that showed comparable oxidation levels in each sample (Figure 4b). It is interesting that M_w_ does not have a large influence on the conversion. Possibly, the gaseous nature of NO_x_ and its excellent solubility in hydrocarbons explain why the M_w_ does not have a large impact on the product yield and distribution.

It is likely that PE waste streams that are subjected to chemical recycling also contain a small fraction of PP. Therefore, the oxidation of PP (M_w_ = 255538 g/mol) with O_2_/NO_x_ was also studied. The GC data (Figure 4c) shows that PP is converted to branched dicarboxylic acids in very small amounts (2 mol%). The observed dicarboxylic acids have a chain length comparable to PE derived diacids and contain the same methyl groups as the backbone of PP. In addition, the carbon recovery was lower compared to the same reaction in PE (48 mol% vs. 81 mol% after 2 h reaction time). Likely, PP and its cleavage products are more easily overoxidized to gaseous products like CO and CO_2_. IR spectra of oxidized PP show that the dominant functional groups are the same as in PE, namely carboxylic acid, nitro, and nitrate (Figure 4d). This suggests that the reaction of PP proceeds through a similar reaction mechanism as PE, starting with a C‐H abstraction. The main difference between the two polymers is that PP has tertiary carbon atoms. As a consequence, PP oxidation proceeds at a faster rate and the branched dicarboxylic acids products have a lower stability under reaction conditions, making them more susceptible to over‐oxidation.

The effect of impurities was studied by comparing above results obtained with virgin polymers to what is obtained with a waste polymer (DKR 310) with a quality that could end up in chemical recycling facilities (Figure 4e). This waste fraction was cleaned with hot water, and consisted of a mixture of PE and PP (5:1) [28]. The ash content of the cleaned sample is 5.27 % and several impurities were still present (e.g., Ti, Ca, Si, Fe, Cu, Al, and Cl). The polyolefin waste was subjected to oxidative conditions (i.e., 120°C, 4 h, P_NO_ = 4 bar, and P_O2_ = 8 bar) and an oily substance was formed that is similar to the product obtained with virgin HDPE (Figure 4f). The mixed polyolefin waste yields a lower yield of linear dicarboxylic acid of 20 mol% compared to 25 mol% obtained from the virgin material (Figure 4g). This difference can be explained by the presence of PP, which does not contribute to the linear dicarboxylic acid yield. CHN analysis (Figure 4g) showed a comparable carbon recovery (63 mol% vs. 64 mol%), and IR spectroscopy analysis (Section S4) indicates the same main reaction products. Overall, it can be concluded that the oxidative conversion of PE with O_2_/NO is not significantly impacted by the impurities present in post‐consumer waste, including PP. However, PP itself does not contribute to the value of the product stream, as most is converted to gaseous products (CO/CO_2_).

### 2.3 Effect of Catalyst Addition

**FIGURE 5:**
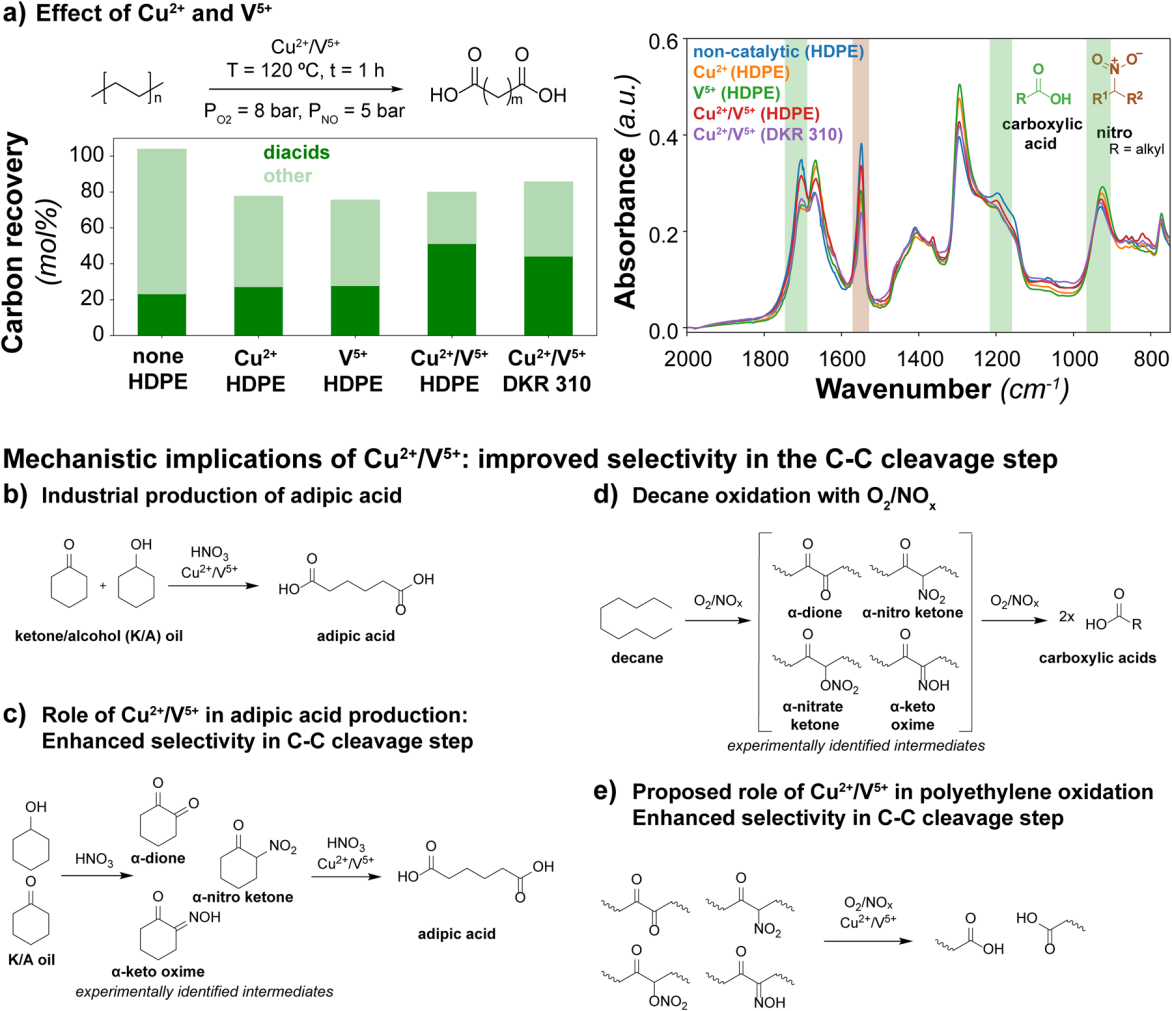
(a) Carbon recovery of the oil fraction (light green), dicarboxylic acid (dark green) yield and infrared (IR) spectra from the conversion of high‐density polyethylene (HDPE) and DKR 310. The polymers were oxidized at 120°C for 4 h with PNO = 5 bar and PO2 = 8 bar. (b) Industrial production of adipic acid. (c) Role of Cu^2+^/V^5+^ in adipic acid production. (d) Decane oxidation with O_2_/NO_x_ proceeds through α‐oxidized ketone intermediates. (e) Proposed role of Cu^2+^/V^5+^ in polyethylene oxidation.

The primary industrial route for adipic acid production is the oxidation of cyclohexanone/cyclohexanol (K/A oil) with HNO_3_ (Figure 5b) [29]. This process employs Cu^2^^+^ and V^5^^+^ catalysts, typically introduced as Cu(NO_3_)_2_ and NH_4_VO_3_. During the oxidation of K/A oil with HNO_3_, several more highly oxidized cyclohexanone derivatives have been identified, including 1,2‐cyclohexanedione, 2‐nitrocyclohexanone, and 2‐oximecyclohexanone [21, 30]. Both Cu^2^^+^ and V^5^^+^ are believed to play a crucial role in the selective decomposition of these intermediates to adipic acid. Although K/A oil oxidation with HNO_3_ can proceed in the absence of transition metal‐based catalysts, the selectivity is typically significantly lower, due to unselective decomposition pathways of these intermediates. Studies using α‐functionalized cyclohexanone model compounds have shown that these cyclic intermediates can be converted to adipic acid with high selectivity (∼95%) in the presence of Cu^2^^+^/V^5^^+^ catalysts (Figure 5c), while their selectivity is markedly lower in the absence of catalyst [21, 30].

In a detailed mechanistic study on the oxidation of decane to carboxylic acids using O_2_/NO_x_, it was shown that the reaction proceeds via α‐oxidized ketone intermediates prior to C─C bond cleavage (Figure 5d) [20]. The experimentally observed intermediates included decane derivatives bearing α‐dione, α‐nitro ketone, α‐nitrate ketone, and α‐keto oxime functionalities. The presence of these species, combined with the fact that similar oxidants (NO, NO_2_, HNO_2_, and HNO_3_) are present in both reaction systems, albeit in different ratios, highlights the close resemblance between the two reaction pathways. Therefore, we hypothesize that the yield of dicarboxylic acids in PE oxidation with O_2_/NO_x_ can be significantly enhanced by the addition of a Cu^2^^+^/V^5^^+^ catalyst.

The polymer was subjected to optimal oxidative conditions (120°C, 1 h, P_NO_ = 5 bar, and P_O2_ = 8 bar) in the presence of 1 mol% Cu^2^^+^/V^5^^+^ catalyst with respect to the number of methylene units (added as Cu(NO_3_)_2_ and NH_4_VO_3_). Under these conditions, the dicarboxylic acids yield increased to ∼51 mol%, compared to ∼23 mol% without catalyst (Figure 5a). At the same time, a carbon recovery of ∼80 mol% was maintained in the oil phase. When applied individually, either Cu^2^^+^ or V^5^^+^ increased the dicarboxylic acids yield only up to ∼27 mol%, demonstrating that both metals must act in concert to achieve the higher yield. IR spectroscopy did not reveal clear differences between samples with and without catalyst, suggesting that both metal ions interact with reactive intermediates that are present only in low concentrations. These intermediates are most likely α‐diones, α‐nitro ketones, and α‐nitrate ketones, as reported in previous studies [20]. Although the exact mechanism of adipic acid formation in HNO_3_‐based oxidation remains unresolved in the academic literature, hypotheses can be formulated based on studies of metal‐catalyzed oxidative C─C bond cleavage [29, 31]. In particular, α‐diones have been investigated in more detail and are known to act as chelating ligands toward transition metal ions. It is likely that other α‐functionalized ketones can coordinate in a similar manner. These coordinated species may then undergo selective C─C bond cleavage, yielding two carboxylic acids. We therefore conclude that the Cu^2^^+^/V^5^^+^ catalyst improves the selectivity in the C─C cleavage step, leading to a higher yield toward dicarboxylic acids (Figure 5e).

Finally, the waste polyolefin sample (DKR 310) was subjected to oxidative conditions in the presence of Cu^2^^+^/V^5^^+^. The resulting dicarboxylic acid yield (∼43 mol%) and carbon recovery (∼86 mol%) were comparable to those obtained with virgin HDPE (Figure 5a). This demonstrates that the Cu^2^^+^/V^5^^+^ catalytic system is not affected by the impurities present in post‐consumer plastics, highlighting its robustness for the conversion of real‐life plastic recycling feeds into dicarboxylic acids.

## 3 Conclusion

The oxidation of polyethylene was studied as a function of reaction parameters, the presence of a Cu^2+^/V^5+^ catalyst, and polymer properties. Among the variables tested, NO pressure proved most critical, directly enhancing reaction rate, diacid yield, and carbon recovery. O_2_ pressure primarily influenced the reaction rate, supporting the view that O_2_ serves to regenerate reduced NO_x_ species, while the actual oxidation of PE occurs via NO_x_ species. Without catalyst, the selection of optimized reaction conditions afforded yields toward dicarboxylic acids of up to ∼29 mol%. Furthermore, the introduction of a Cu^2^^+^/V^5^^+^ catalytic system significantly improved the performance, delivering dicarboxylic acids yields up to ∼51 mol%, while maintaining high carbon recovery (∼80 mol%). Similar selectivity and dicarboxylic acid yield were obtained for HDPE, LDPE, LLDPE, Low M_w_ LDPE and heavily contaminated post‐consumer waste, underlining the robustness of the developed catalytic conversion process. The high carbon recovery of the route under high NO pressure paves the way for further optimization by reactor engineering. Future work should focus on reactor engineering and product separation strategies to further increase overall yields through multiple oxidation cycles.

## 4 Experimental Section

### 4.1 Chemicals and Materials

The following chemicals were used: acetyl chloride (Sigma Aldrich, >99%), methanol (Sigma Aldrich, >99%), ammonium metavanadate (Sigma Aldrich, 99%), copper (II) nitrate trihydrate (Sigma Aldrich, 99–104%) and 4‐heptanone (Acros Organics, >98%). All chemicals were used as received.

All polymer properties mentioned below were provided by the supplier, except the melting point (T_m_) and the gel permeation chromatography (GPC) analysis which was executed at Sabic. The GPC measurements at Sabic were performed on a Freeslate Rapid GPC that contains two Plgel 10 µM Mixed B columns in series. The GPC was calibrated using the Polystyrene (PS) calibration kit from Agilent (PL2010‐0100). The calibration kit contained 10 different individual PS molar mass standard with a peak molecular weight (M_p_) ranging from 580 to 3 000 000 g/mol. The low mass average molecular weight (M_w_) polyethylene (PE, Sigma Aldrich) material was obtained as powder and had the following properties: M_w_ supplier = 4000 g/mol, number average molecular weight (M_n_) supplier = 1700 g/mol, T_m_ = 98°C, density (*ρ*) = 0.92 g/cm^3^, M_w_ = 4097 g/mol and M_n_ = 1805 g/mol. The low‐density polyethylene (LDPE, Lupolen 3020H) was obtained from LyondellBasell as pellets and had the following properties: T_m_ = 114°C, ρ = 0.928 g/cm^3^, melt flow index 190°C/2.16 kg (MFI) = 2 g/10 min, M_w_ = 80 980 g/mol and M_n_ = 23 755 g/mol. The high‐density polyethylene (HDPE) was obtained from Sabic as powder and had the following properties: M_w_ = 137 500 g/mol, M_n_ = 16 010 g/mol and T_m_ = 114°C. The linear low‐density polyethylene (LLDPE) was obtained from Sigma Aldrich as pellets and had the following properties: mp = 124°C, MFI 190°C/2.16 kg = 100 g/10 min, M_w_ = 52 021 g/mol and M_n_ = 12 559 g/mol. The polypropylene (PP) was obtained from Sigma Aldrich as pellets and had the following properties: M_n_ = 61766, M_w_ = 255538 g/mol, MFI 230°C/2.16 kg = 12 g/10 min, ρ = 0.9, and T_m_ = 160°C–165°C. The PE waste sample (DKR 310) was obtained as pellets from TNO (Petten, The Netherlands) and had a PE/polypropylene (PP) ratio of approximately 5. The sample had a total ash content of 5.17 wt.% and contained the following impurities: Cl (123 ppm), F (58 ppm), S (273 ppm), Al (662 ppm), Ba (595 ppm), Ca (12013 ppm), Cd (6 ppm), Cr (19 ppm), Cu (22 ppm), Fe (411 ppm), K (113 ppm), Mg (363 ppm), Mn (8 ppm), Na (99 ppm), Ni (6 ppm), P (133 ppm), Pb (54 ppm), Si (1795 ppm), Sr (14 ppm), Ti (7572 ppm), and Zn (77 ppm).

### 4.2 Reactor Experiments

The reactor experiments were performed batchwise in a Parr autoclave reactor of 50 mL (+7 mL in headspace). In each experiment, a borosilicate glass liner was filled with 200 mg PE and the glass liner was placed in the reactor. The experiments with catalyst (Cu(NO_3_)_2_.3H_2_O and NH_4_VO_3_) were performed with 1 mol% metal with respect to the number of methylene units in the polymer. As the experiments were non‐stirred, the metals were mixed with the polymer materials as homogeneously as possible before the glass liner was placed in the reactor.

Prior to the oxidation experiment, the reactor was purged with N_2_. Then, the vessel was filled with NO (Linde gas, 20% in N_2_) to the desired pressure, followed by the desired O_2_ (Linde gas, 40% in N_2_) and N_2_ (Linde gas) pressure. The pressures are reported at room temperature. The PE layer in the bottom of the reactor was too thin to apply stirring. Subsequently, the autoclave was heated according to the desired temperature program. The reactor needs 1 h to reach the final temperature. This timepoint was taken as the start of the reaction. After the desired temperature program was completed, the reactor was allowed to cool down to room temperature. To ensure reproducibility Ni paste needs to be avoided as sealant. Possibly Ni leads to the dissociative adsorption of NO_x_ on Ni, leading to a wide variation in reaction results, especially with a low NO pressure. Therefore, Teflon tape was used as sealant.

### 4.3 Product Workup and Analysis

Prior to product analysis with gas chromatography (GC), about half of the crude reaction mixture was esterified with a mixture of 6 mL acetyl chloride/methanol (1:1; v:v) at 50°C for 1 h. The other half of the sample was kept aside for infrared (IR) spectroscopy and CHN analysis. In most experiments, the esterified solution is clear and slightly yellow. Subsequently, one droplet (∼10 mg) of 4‐heptanone was added as internal standard (the exact weight was used for determining the yield). To prevent clogging of the GC, the sample was filtered with a 0.45 µm syringe filter. Weighing the filter revealed no detectable amount of residual.

Gas chromatography (GC) analysis on oxidized PE samples was performed on a GC x GC (Shimadzu, GC‐2010 Plus) coupled to a flame ionization detector (FID) and mass spectrometer (MS) (Shimadzu, GCMS‐QP2010 Ultra). Samples were injected at an injector temperature of 200°C. The products were separated using a GC 60 m, 0.25 mm ID, 0.25 µm column (separation on boiling point) and a VF‐5 ms, 30 m, 0.25 ID, 0.25 µm column (separation on polarity). The column oven was kept at 40°C for 5 min, increased at 5°C/min to 280°C and held for 10 min. In the chromatograms, the peaks were assigned based on the GC‐MS fragmentation patterns, while a quantitative analysis was performed with the help of an internal standard in GC‐FID. Response factors for GC‐FID were obtained by using effective carbon number (ECN) theory [23, 32] The yields are defined in the percentage of carbon that ends up in each product (Equation 1). Experiments under optimal conditions were repeated several times (e.g., optimal non‐catalytic, optimal catalytic, and experiments with DKR 310), showing a typical variation in dicarboxylic acid yield up to ±10% on the reported number. The average numbers are given in the paper.

(1) $$\mathrm{Yield}\;\left(\mathrm{mol}\%\right)=\frac{C_{\mathrm{diacids}}}{C_{\mathrm{PE}}}\;\times 100\%$$

IR spectra of the crude reaction mixture were measured in attenuated total reflectance (ATR) mode on a Perkin Elmer FT‐IR Frontier spectrometer with mercury, cadmium, and telluride (MCT) detector, using a Perkin Elmer Universal ATR sampling accessory.

For CHN analysis, the crude reaction mixtures were submitted to MEDAC Ltd. The results were used as received and the amount of recovered carbon in the oil fraction was calculated by multiplying the percentage of carbon obtained with CHN analysis times the sample weight of the crude reaction mixture (Equation 2). The batch‐to‐batch variation in the carbon recovery determined with CHN analysis is up to ∼5%.

(2) $$\begin{array}{cc} \mathrm{Carbon}\;\mathrm{recovery}\;\left(\%\right) & =\frac{C_{\mathrm{oil}\;\mathrm{fraction}}}{C_{\mathrm{PE}\;\mathrm{start}\;\mathrm{material}}}\;\times 100\%\;\\ & =\frac{\mathrm{weight}\;\mathrm{oil}\;\mathrm{fraction}*\%C\;}{C_{\mathrm{PE}\;\mathrm{start}\;\mathrm{material}}}\;\times 100\% \end{array}$$

IR spectra of the gas phase were recorded on a Bruker Tensor 37 spectrometer. When the autoclave was cooled down to room temperature, the gas was transferred in a gas bag to a transmission IR cell (transmission path = 1 cm). It was observed that there is an increase in NO_x_ concentration toward the fraction removed last from the reactor. Upon transferring the gas to the gas bag, the color of the exhaust gas changed from colorless to dark orange. As a consequence, only a qualitative analysis could be performed. Before the measurement, the cell and measurement chamber were flushed with a N_2_ flow. When a stable background signal was reached without CO_2_ signal, a background was recorded. Then, the contents of the gas bag collected from the reactor were introduced into the IR cell using a flow meter. Spectra were recorded after the signal stabilized. For spectral analysis, the background signal collected under N_2_ was subtracted to yield a difference spectrum.

Differential scanning calorimetry (DSC) measurements were performed on a TA instruments DSC using the following temperature program: heating from 0°C to 220°C with 10°C/min and then keeping the sample at 220°C for 3 min. Subsequently, the sample was cooled to 0°C with a ramp of 10°C/min and kept at 0°C for 3 min. The second heating cycle was performed with a temperature ramp of 10°C/min to 220°C. The melting temperatures of the first heating cycle were used in the paper.

## Author Contributions

**Tom J. Smak**: conceptualization, methodology, investigation, data curation, validation, formal analysis, writing – original draft, writing – review and editing, and visualization. **Rinke Altink**: writing – review and editing, supervision, and conceptualization. **Ina Vollmer**: conceptualization, supervision, and writing – review and editing. **Bert M. Weckhuysen**: conceptualization, funding acquisition, writing – review and editing, supervision, resources, and project administration.

## Conflicts of Interest

The authors declare no conflicts of interest.

## Supporting information




**Supporting File**: The authors have cited additional references within the Supporting Information [35, 36, 37, 38, 39, 40, 41, 42, 43, 44, 45, 46, 47, 48, 49, 50, 51, 52, 53]. The file also contains an extended section on prior art and additional IR spectra of the gas phase, the effect of NO pressure, and oxidized post‐consumer waste.

## Data Availability

All data and python scripts utilized in the manuscript have been uploaded to the YODA repository and are available under https://doi.org/10.24416/UU01‐AURM7T.
