# Supplementary material for: Enhanced Formation of Dicarboxylic Acids in the Catalytic Oxidation of Polyethylene With O2 and NOx
Source: Angew Chem Int Ed Engl. 2026 Jun 26;65(34):e6681773. doi: 10.1002/anie.6681773 (PMC13480809; doi:10.1002/anie.6681773)
Supplement: Supplementary file 1 [file ANIE-65-e6681773-s001.docx]

Supporting Information

**Enhanced Formation of Dicarboxylic Acids in the Catalytic Oxidation of Polyethylene with O_2_ and NO_x_**

Tom J. Smak^a^, Rinke Altink^b^, Ina Vollmer^a,*^ and Bert M. Weckhuysen^a,*^

a) Inorganic Chemistry and Catalysis group, Institute for Sustainable and Circular Chemistry, Department of Chemistry, Faculty of Science, Utrecht University, Universiteitsweg 99, 3584 CG Utrecht (The Netherlands)

b) TNO Brightsite, Urmonderbaan 22, 6167 RD Geleen (The Netherlands)

* To whom correspondence should be addressed; [b.m.weckhuysen@uu.nl](mailto:b.m.weckhuysen@uu.nl) and [i.vollmer@uu.nl](mailto:i.vollmer@uu.nl)

**Table of Contents**

[**1.**	**Prior Art on Polyethylene Oxidation towards Dicarboxylic Acids**	3](#_Toc229859009)

[**2.**	**Gas-phase Infrared Spectra**	6](#_Toc229859010)

[**3.**	**Additional Data on the Effect of NO Pressure**	6](#_Toc229859011)

[**4.**	**Infrared Spectra of Real Plastic Waste**	7](#_Toc229859012)

[**5.**	**DSC Graphs of the Polyolefin Starting Materials**	7](#_Toc229859013)

[**6.** **Replicates of the GC-FID Graphs with Cu^2+^/V^5+^**	8](#_Toc229859014)

[**7.**	**References**	8](#_Toc229859015)

# 1. **Prior Art on Polyethylene Oxidation towards Dicarboxylic Acids**

Polyethylene (PE) oxidation is extensively studied in the academic literature and goes back many decades. In the past, the majority of the studies focuses on oxidation events from a polymer degradation perspective, aiming to prevent it. The reaction parameters that are studied involve the gas atmosphere (mostly air), temperature and UV-exposure aiming to simulate weathering conditions, albeit in accelerated form.^1–6^ For weathering purposes, O_2_ (air) is by far the most common oxidant, but stronger oxidants, such as O_3_,^7^ NO_2_^8^ and HNO_3_^9^, also have been reported. It has been shown that upon oxidation of PE a broad range of functional groups can be introduced into the PE backbone, such as alcohols,^10,11^ ketones,^11,12^ oximes^13,14^ and nitro^15^ groups. Polymer degradation is often studied with analytical techniques, such as infrared (IR) spectroscopy, differential scanning calorimetry (DSC) and nuclear magnetic resonance (NMR). In 1998, Pifer and Sen were the first to report that PE can be converted into well-defined short-chain dicarboxylic acid products, such as succinic and adipic acid.^16^ More examples followed and several research groups started to study small cleavage products with analytical methods, such as gas chromatography (GC) and high pressure liquid chromatography (HPLC).

A complete overview of the strategies to convert PE into well-defined dicarboxylic acids is shown in **Figure S1**. A direct comparison, between all the reported methods is challenging due to the variation of the methods and how the product yield is reported. Often product yields are reported based on wt.% instead of mol%. In addition, other factors, such as normalization protocols, make a direct comparison difficult. In order to make a more fair comparison, the product yields have been converted from wt.% to mol% based yields for some papers. The mol% based yield is defined as the percentage of carbon from the original PE material that is retained in the dicarboxylic acid products.

Dicarboxylic acid products can already be produced from PE in 7.5 mol% yield, when it is exposed to 30 bar of synthetic air at 130 °C under neat conditions for 16 h (**Figure S1a**) and in the absence of a catalyst.^17^ In addition to carboxylic acids, a broad range of other functional groups was observed, such as esters, γ‑lactones and γ-ketones.

Davydovich *et al.* reported a method to improve the yield towards dicarboxylic acids up to ~ 12 mol% with the addition of a CuSO_4_ catalyst (**Figure S1b**).^18^ The reactions were performed in an aqueous system at 130 – 150 °C, with 30 – 100 psi O_2_ for 20 h. Wang *et al.* reported the non-catalytic conversion of polyethylene into dicarboxylic acids under aqueous conditions at 160 °C for 6 h with 1 MPa O₂ pressure. Linear dicarboxylic acids (C₄–C₁₀) were obtained in yields of up to 72 mol%, with only minor formation of other products.^19^ A more recent study described a photothermal oxidative conversion under aqueous conditions at 130 °C and 1.5 MPa air for 1.5 h, using a Pt/TiO₂ catalyst under light irradiation (370 mW cm⁻²). In this case, a dicarboxylic acid-containing oil was obtained in 84 wt% yield. However, detailed characterization of the product mixture was limited, as no quantitative GC analysis was performed to determine the exact composition of the dicarboxylic acids. Therefore, this example is not included in **Figure S1**.^20^

Promising results for the aerobic oxidation of PE were obtained under conditions similar to the *Amoco Mid-Century* technology (**Figure S1c**). In 2003, Partenheimer was the first to explore this chemistry for PE oxidation.^21,22^ Using a Co/Mn/Zr/Br catalytic system with 70 bar air at 150 °C in acetic acid they were able to obtain succinic, glutaric and adipic acid in a combined yield of up to 46 mol%. Later, similar conditions and yields were reported by Sullivan *et al.* who demonstrated that the method also works for a mixed feedstock of HDPE, PET and PS.^23^ In addition, they demonstrated that with the use of engineered bacteria the mixed product obtained from oxidation can be funneled to either β‑ketoadipate or polyhydroxyalkanoates.^23^ In 2024, the start-up *Radical Dot* was founded, which aims to develop the Co/Mn/Br chemistry further for mixed plastics and bring it to the market.

Another cobalt-based oxidative method was reported by Li *et al.*, who used a spent lithium cobalt oxide cathode as catalyst material (**Figure S1d**). The reaction proceeded at 130 °C with 10 bar O_2_ for 6 h. Li *et al.* obtained a dicarboxylic acid yield up to 77.5 wt.%, with dicarboxylic acids with a median chain length of ~ 5 carbon atoms, which translates to a yield of ~ 42 mol%.^24^

Sen and co-workers reported an oxidative method to convert PE into carboxylic acids in yields up to ~ 39 mol%.^16,25^ Their method, involves the oxidation under neat conditions at 170 °C in the presence of O_2_ and NO (**Figure S1e**). The product mixture consists of short-chain dicarboxylic acids, succinic, glutaric, adipic and pimelic acid. Later a similar procedure was mentioned in a patent by Yao *et al.*, albeit at milder conditions, but the product mixture was similar in terms of yield and composition.^26^ The use of aerobic NO_x_ is attractive, because it allows to work under neat conditions and in theory O_2_ is still used as the terminal oxidant. The chemistry of aerobic NO_x_ is still largely unexplored and both reports do not discuss the reaction mechanism and the reaction parameters influencing the outcome.

A strategy somewhat comparable to aerobic NO_x_ is the use of stoichiometric HNO_3_ (**Figure S1f**). Although it has been known for a long time that carboxylic acid groups form upon oxidation of PE with HNO_3_, Bäckström *et al.* was the first to demonstrate that PE can be converted to mixtures of succinic, glutaric, adipic and pimelic acid with stoichiometric amounts of HNO_3_.^27^ Later, similar observations were made by Pinsuan *et al.*^28^ and Yao *et al*.^26^ These methods involving stoichiometric HNO_3_ are commercially most advanced, with *Novoloop* successfully scaling up to a pilot plant in 2024.

Last there are two recent examples that report very high dicarboxylic acids yields, which are not included in the scheme below. In 2023, Wang *et al.* has reported a method to depolymerize PE into low molecular weight aliphatic dicarboxylic acids with ~ 85 % yield in the presence of a Ru/TiO_2_ catalyst at 160 °C in H_2_O after 24 h reaction time.^29^ Although they define the yield as amount of carbon present in the dicarboxylic acid product, it is uncertain how they obtained this number, because the manuscript does not include a method that can be used to determine this number. GPC, IR and NMR (without internal standard) were used for product analysis. In addition, their GPC data suggest that they mainly have oligomeric species with a M_w_ of 1300 g/mol and in our opinion the reported NMR data suggests that a variety of other functional groups is still present in the sample. Building forward on the work of Wang *et al.*, Zhang *et al.*^30^ has reported a method to convert PE into dicarboxylic acids with a yield of ~ 86 %. The reported method involves oxidation under neat conditions in the presence of a Co-MCM-41 catalyst with 10 bar O_2_. Unfortunately, it is not fully clear how to interpret their findings, due to some contradictions in the reported data. For example, one of the main conclusions is that dicarboxylic acids ranging from C4 to C24 are produced, while simultaneously the GPC data shows a M_w_ of several thousands, suggesting that not everything is observed with GC. Furthermore, in the NMR data the aliphatic signal (-C**H_2_**‑) has a comparable integral as the α-carboxyl signal (‑C**H_2_**‑COOH), suggesting only very short dicarboxylic acids, which does not match the result obtained with GC and GPC.

**Figure S1.** Prior art on oxidative chemical recycling methods of polyethylene (PE). (a) Aerobic auto-oxidation of PE. (b) Aerobic oxidation under aqueous conditions (c) Methods involving conditions comparable to the Amoco-Mid Century technology. (d) Methods involving LiCo_2_ catalyst. (e) Methods involving O_2_/NO. (f) Methods involving a stoichiometric amount of HNO_3_.

# 2. **Gas-phase Infrared Spectra**

**Figure S2.** (a) Infrared (IR) spectrum of the gas phase obtained from a sample taken from reaction before heating (P_NO_ = 4 bar, and P_O2_ = 8 bar). (b) IR spectrum of the gas phase obtained from a sample taken after the reaction (120 °C, 4 h, P_NO_ = 4 bar, and P_O2_ = 8 bar).

# 3. **Additional Data on the Effect of NO Pressure**

**Figure S3.** Dicarboxylic acid yield, carbon recovery and infrared (IR) spectra of oxidized high-density polyethylene (HDPE) as a function of NO pressure. HDPE was oxidized for 4 h at 120 °C with the following gas pressures, P_O2_ = 8 bar, balance and N_2_ P_total_ = 50 bar.

# 4. **Infrared Spectra of Real Plastic Waste**

**Figure S4**. Infrared (IR) spectra of high-density polyethylene (HDPE) and mixed polyolefin waste oxidized for 4 h at 120 °C with the following gas pressures, P_O2_ = 8 bar, P_NO_ = 4 bar, balance N_2_ and P_total_ = 50 bar.

# 5. **DSC Graphs of the Polyolefin Starting Materials**

**Figure S5.** Differential scanning colorimetry (DSC) curves (exo up) of the polyolefin start materials used in this study. The following polymer materials were used: high-density polyethylene (HDPE) (a), low-density polyethylene (LDPE) (b), linear low-density polyethylene (LLDPE) (c), low molecular weight (M_w_) LDPE (d), polypropylene (PP) (e), and DKR 310 (f).

# **6. Replicates of the GC-FID Graphs with Cu^2+^/V^5+^**

**Figure S6.** Gas chromatography-flame ionization detection (GC-FID) chromatograms of high-density polyethylene (HDPE) (a/b/c) and DKR 310 (d/e) oxidized at 120 °C for 4 h with P_NO_ = 5 bar and P_O2_ = 8 bar with 1 mol% C in the presence of 1 mol% Cu²⁺/V⁵⁺ catalyst with respect to the number of methylene units (added as Cu(NO_3_)_2_ and NH_4_VO_3_). The following dicarboxylic acid yields were obtained, 49 (a), 51 (b), 55 (c), 45 (d) and 41 (e) mol%.

# 7. **References**

(1)	Grause, G.; Chien, M. F.; Inoue, C. Changes during the Weathering of Polyolefins. *Polym. Degrad. Stab.* **2020**, *181*, 109364. https://doi.org/10.1016/j.polymdegradstab.2020.109364.

(2)	Gijsman, P. Review on the Thermo-Oxidative Degradation of Polymers during Processing and in Service. *E-Polymers* **2008**, *8*, 65. https://doi.org/10.1515/epoly.2008.8.1.727.

(3)	Gugumus, F. Formation of Ester Functional Groups in Oxidizing Polymers. *Polym. Degrad. Stab.* **1999**, *65*, 5–13. https://doi.org/10.1016/S0141-3910(98)00208-0.

(4)	Gugumus, F. Re-Examination of the Thermal Oxidation Reactions of Polymers 2. Thermal Oxidation of Polyethylene. *Polym. Degrad. Stab.* **2002**, *76*, 329–340. https://doi.org/10.1016/S0141-3910(02)00029-0.

(5)	Gugumus, F. Re-Examination of the Thermal Oxidation Reactions of Polymers 3. Various Reactions in Polyethylene and Polypropylene. *Polym. Degrad. Stab.* **2002**, *77*, 147–155. https://doi.org/10.1016/S0141-3910(02)00093-9.

(6)	Rugg, F. M.; Smith, J. J.; Bacon, R. C. Infrared Spectrophotometric Studies on Polyethylene. II. Oxidation. *J. Polym. Sci.* **1954**, *13*, 535–547. https://doi.org/10.1002/pol.1954.120137202.

(7)	Beachell, H. C.; Nemphos, S. P. Oxidative Degradation of Polyethylene. *J. Polym. Sci.* **1956**, *21*, 113–124. https://doi.org/10.1002/app.1972.070161006.

(8)	Ogihara, T. Oxidative Degradation of Polyethylene in Nitrogen Dioxide. *Bull. Chem. Soc. Jpn.* **1963**, *36*, 58–63. https://doi.org/10.1246/bcsj.36.58.

(9)	Melby, L. R. Nitric Acid Oxidation of High-Density Polyethylene. Organic Chemical Aspects. *Macromolecules* **1978**, *11*, 50–56. https://doi.org/10.1021/ma60061a010.

(10)	Bunescu, A.; Lee, S.; Li, Q.; Hartwig, J. F. Catalytic Hydroxylation of Polyethylenes. *ACS Cent. Sci.* **2017**, *3*, 895–903. https://doi.org/10.1021/acscentsci.7b00255.

(11)	Chen, L.; Malollari, K. G.; Uliana, A.; Sanchez, D.; Messersmith, P. B.; Hartwig, J. F. Selective, Catalytic Oxidations of C–H Bonds in Polyethylenes Produce Functional Materials with Enhanced Adhesion. *Chem* **2021**, *7*, 137–145. https://doi.org/10.1016/j.chempr.2020.11.020.

(12)	Teo, J. Y. Q.; Yeung, C. W. S.; Tan, T. T. Y.; Loh, W. W.; Loh, X. J.; Lim, J. Y. C. Benzaldehyde-Mediated Selective Aerobic Polyethylene Functionalisation with Isolated Backbone Ketones. *Green Chem.* **2022**, *24*, 6287–6294. https://doi.org/10.1039/d2gc02502g.

(13)	Lemmens, R.; Vercammen, J.; Van Belleghem, L.; De Vos, D. Upcycling Polyethylene into Closed-Loop Recyclable Polymers through Titanosilicate Catalyzed C-H Oxidation and in-Chain Heteroatom Insertion. *Nat. Commun.* **2024**, *15*, 9188. https://doi.org/10.1038/s41467-024-53506-9.

(14)	Otten, M.; Klein Gebbink, I.; Schara, P. J.; Tomovic´, Z.; Lutz, M.; Bruijnincx, P. C. A.; Thevenon, A. Post-Polymerization Modification of Polyethylene through Photochemical Oximation and Consecutive Ketonization. *J. Am. Chem. Soc.* **2025**, *147*, 22827–22838. https://doi.org/10.1021/jacs.5c05212.

(15)	Wei, X.; Shen, C.; Ye, P.; Liu, X.; Xu, S.; Wang, Y. Z. Highly Adaptable Oxidative Upcycling of Polyolefins to Multifunctional Chemicals Containing Oxygen and Nitrogen. *Mater. Horizons* **2025**, *12*, 4388. https://doi.org/10.1039/d5mh00132c.

(16)	Pifer, A.; Sen, A. Chemical Recycling of Plastics to Useful Organic Compounds by Oxidative Degradation. *Angew. Chem. Int. Ed.* **1998**, *37*, 3306–3308. https://doi.org/10.1002/(SICI)1521-3773(19981217)37:23<3306::AID-ANIE3306>3.0.CO;2-B.

(17)	Smak, T. J.; de Peinder, P.; Van der Waal, J. C.; Altink, R.; Vollmer, I.; Weckhuysen, B. M. Oxidative Conversion of Polyethylene Towards Di-Carboxylic Acids: A Multi-Analytical Approach. *ChemSusChem* **2024**, *17*, e202301198. https://doi.org/10.1002/cssc.202301198.

(18)	Davydovich, O.; Choudhary, H.; Martinez, D. V.; Salinas, J. E.; Martinez, E. J.; Davis, R. D.; Bays, N. R.; Schafer, D. P.; Kent, M. S. Mild Aqueous Metal Catalyzed Oxidative Conversion of Low-Density Polyethylene to Low Molecular Weight Aliphatic Carboxylic Acids. *Green Chem.* **2024**, *26*, 10390–10396. https://doi.org/10.1039/d4gc02187h.

(19)	Chang, Y.; Gao, W.; Wang, S.; Zhu, B.; Zhao, K.; Hu, J.; Lim, K. H.; Liu, P.; Wang, W. J.; Li, B. G.; Wang, Q. Aqua-Oxidation of Polyethylene Into Carboxylic Acids Under Mild Conditions: A Catalyst-Free Upcycling Strategy for Nonpolar Plastics. *Angew. Chem. Int. Ed.* **2026**, *138*, e23067. https://doi.org/10.1002/anie.202523067.

(20)	Wang, K.; Hao, Y.; Cheng, C.; Yuan, F.; Zhu, Y.; Yan, J.; Jia, R.; Cheng, P.; Yang, J.; Shi, L.; Huang, L.; Wang, X. Photothermal Oxidative Upcycling of Polyethylene to Aliphatic Dicarboxylic Acids over a Pt/TiO_2_ Catalyst. *ACS Catal.* **2026**, *16*, 1929–1935. https://doi.org/10.1021/acscatal.5c08019.

(21)	Partenheimer, W. Valuable Oxygenates by Aerobic Oxidation of Polymers Using Metal / Bromide Homogeneous Catalysts. *Catal. Today* **2003**, *81*, 117–135. https://doi.org/10.1016/S0920-5861(03)00124-X.

(22)	Partenheimer, W. Transformation of Polymers to Useful Chemical Oxidation. US 6,958,373 B2, 2005.

(23)	Sullivan, A. K. P.; Werner, A. Z.; Ramirez, K. J.; Ellis, L. D.; Bussard, J.; Black, B. A.; Brandner, D. G.; Bratti, F.; Buss, B. L.; Dong, X.; Haugen, S. J.; Ingraham, M. A.; Konev, M. O.; William, E.; Miscall, J.; Pardo, I.; Woodworth, S. P.; Guss, A. M.; Román-, Y.; Stahl, S. S.; Beckham, G. T. Mixed Plastics Waste Valorization through Tandem Chemical Oxidation and Biological Funneling. *Science,* **2022**, *378*, 207–211. https://doi.org/10.1126/science.abo4626.

(24)	Li, S.; Feng, Q.; Li, Q.; Xie, Y.; Xu, P.; Wang, Z. Synergistic Co-Recycling : Selective Oxidation of Polyethylene to Dicarboxylic Acids over Spent LiCoO_2_ Cathodes. *Angew. Chem. Int. Ed.* **2025**, *64*, e202501509. https://doi.org/10.1002/anie.202501509.

(25)	Remias, J. E.; Pavlosky, T. A.; Sen, A. Oxidative Chemical Recycling of Polyethene. *Acad. Sci. Paris* **2000**, *3*, 627–629. https://doi.org/https://doi.org/10.1016/S1387-1609(00)01144-0.

(26)	Yun Yao, J.; Wen Wang, Y.; Muppaneni, T.; Shrestha, R.; Le Roy, J.; Figuly, G. D. Products from the Decomposition of Plastic Waste. US10519292 B2, 2019.

(27)	Bäckström, E.; Odelius, K.; Hakkarainen, M. Trash to Treasure: Microwave-Assisted Conversion of Polyethylene to Functional Chemicals. *Ind. Eng. Chem. Res.* **2017**, *56*, 14814–14821. https://doi.org/10.1021/acs.iecr.7b04091.

(28)	Pinsuwan, K.; Opaprakasit, P.; Petchsuk, A.; Dubas, L.; Opaprakasit, M. Chemical Recycling of High-Density Polyethylene (HDPE) Wastes by Oxidative Degradation to Dicarboxylic Acids and Their Use as Value-Added Curing Agents for Acrylate-Based Materials. *Polym. Degrad. Stab.* **2023**, *210*, 110306. https://doi.org/10.1016/j.polymdegradstab.2023.110306.

(29)	Wang, K.; Jia, R.; Cheng, P.; Shi, L.; Wang, X.; Huang, L. Highly Selective Catalytic Oxi‐upcycling of Polyethylene to Aliphatic Dicarboxylic Acid under a Mild Hydrogen‐Free Process. *Angew. Chem. Int. Ed.* **2023**, *135*, e20230140. https://doi.org/10.1002/ange.202301340.

(30)	Zhang, Q.; He, J.; Wei, X.; Shen, C.; Ye, P.; An, W.; Liu, X.; Li, H.; Xu, S.; Su, Z.; Wang, Y. Oxidative Upcycling of Polyethylene to Long Chain Diacid over Co‐MCM‐41 Catalyst. *Angew. Chem. Int. Ed.* **2024**, *63*, e202407510. https://doi.org/10.1002/ange.202407510.

(31)	Garin, F. Mechanism of NOx Decomposition. Appl. Catal. A Gen. 2001, 222, 183–219.

(32)	Morvai, M.; Palyka, L.; Molnår-Perl, I. Flame Ionization Detector Response Factors Using the Effective Carbon Number Concept in the Quantitative Analysis of Esters. J. Chromatogr. Sci. 1992, 30, 448–452.
